# Automated Quality Assurance for Image‐Guided Radiation Therapy

**DOI:** 10.1120/jacmp.v10i1.2919

**Published:** 2009-01-27

**Authors:** Eduard Schreibmann, Eric Elder, Tim Fox

**Affiliations:** ^1^ Department of Radiation Oncology Emory University School of Medicine Atlanta GA U.S.A.

**Keywords:** CBCT, OBI, Trilogy, QA

## Abstract

The use of image‐guided patient positioning requires fast and reliable Quality Assurance (QA) methods to ensure the megavoltage (MV) treatment beam coincides with the integrated kilovoltage (kV) or volumetric cone‐beam CT (CBCT) imaging and guidance systems. Current QA protocol is based on visually observing deviations of certain features in acquired kV in‐room treatment images such as markers, distances, or HU values from phantom specifications. This is a time‐consuming and subjective task because these features are identified by human operators. The method implemented in this study automated an IGRT QA protocol by using specific image processing algorithms that rigorously detected phantom features and performed all measurements involved in a classical QA protocol. The algorithm was tested on four different IGRT QA phantoms. Image analysis algorithms were able to detect QA features with the same accuracy as the manual approach but significantly faster. All described tests were performed in a single procedure, with acquisition of the images taking approximately 5 minutes, and the automated software analysis taking less than 1 minute. The study showed that the automated image analysis based procedure may be used as a daily QA procedure because it is completely automated and uses a single phantom setup.

PACS numbers: 87.56.Fc

## I. INTRODUCTION

The Onboard Imager (OBI) system (Onboard Imager, Varian Medical Systems, Inc., Palo Alto, CA) is designed to advance image guided radiotherapy (IGRT) by incorporating inter/intra‐fraction changes and setup errors into the treatment planning process. The OBI system is comprised of a kV source and detector mounted perpendicularly on the treatment beam that can acquire kV planar radiographic images at certain gantry angles to determine setup errors based on bony anatomy or radio‐opaque markers. To determine inter‐fraction soft tissue changes, 3D CBCT can be obtained from a large set of kV projected images. In CBCT acquisition mode, the gantry rotates while kV projection images are acquired continuously, and a CBCT is then reconstructed from 600 to 700 projections. Megavoltage (MV) imaging is provided by the EPID detector mounted perpendicularly with the treatment beam. The use of this new technology necessitates a comprehensive quality assurance (QA) program to maintain and monitor system performance characteristics.

The quality assurance protocol checks the alignment of the onboard imager with the linear accelerator's isocenter which ensures the coincidence of the OBI's local image coordinates with the accelerator's dosimetric coordinates system.^(^ ^1^ ^–^ ^5^ ^)^ The dosimetric machine coordinate system is a 3D system positioned at isocenter. The OBI's local image coordinate system is bound to the kV or MV detector and is a 2D local system. Deviation of a series of geometrical OBI parameters (such as detector angle, source‐detector‐isocenter distances) affects the correspondence between the dosimetric and local coordinate systems. This results in an incorrect reproduction of objects from the dosimetric system into the image coordinate system that will lead to incorrect patient positioning. The QA protocol ensures that OBI's geometrical parameters are correct and do not affect the conversion from the dosimetric to the image coordinate system.

**Figure 1:**
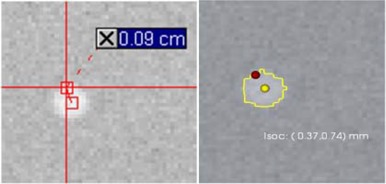
Difference between the automated and manual QA procedure. In the standard procedure, certain distances and locations have to be measured manually, as illustrated in (a) where a marker's center shift from isocenter is assesed using the distance tool. The proposed QA procedure automatically detects marker shape, represented as a yellow line (b). Based on the contour, its center (yellow dot) and its shift from isocenter (red dot) can be automatically assessed.

In the standard QA procedure, a phantom with markers at known locations is placed on the couch and aligned at the isocenter using the room lasers. Then kV, MV and CBCT images of the phantom are obtained using the standard acquisition procedure (Fig. 1), and the location of these markers are compared against phantom specifications to verify the system's ability to correctly position objects in the image according to their location in the treatment room. Yoo et al.^(3)^ used measurements of marker displacements from the digital graticule to asses isocenter shift. Sharpe et al.^(4)^ used image registration tools between a DRRs/planning CT and the OBI‐acquired images to find isocenter displacement. Saw et al.^(1)^ reported on a similar approach. This QA approach used manual identification and measurements with the software tools provided by the vendor – subjective and time consuming tasks that are not suitable for routine monitoring of machine parameters. Technically, an image coordinate system is defined by only two parameters: 1) image origin, and 2) pixel spacing. If correctly calibrated, the OBI's image origin will coincide with the machine's isocenter and the scaling will ensure correct representation of objects' dimensions. In the current QA protocol, different guidelines have been published to ensure correct calibration of an image's origin and spacing.^(2)^ ^,^ ^(3)^ ^,^ ^(5)^ Image origin is checked by measuring deviation in the OBI images of a known marker placed at the machine's isocenter using image registration or the digital graticule. Pixel spacing is verified by comparing distances in OBI‐acquired images against their real world values.

Current QA procedures do not exploit the availability of electronic images. While using the acquired images in digital format, the measurements and philosophy are reminiscent of the period when direct measurements were performed manually on hardcopy films. The checks involved in the standard protocol are implemented by manual identification and measurements of objects in the OBI images; thus, they are subjective time‐consuming tasks not suitable for routine monitoring of machine parameters. This is illustrated in Fig. 1 where the shift from the isocenter is assessed using the distance measurement tool. To eliminate this time‐consuming task and improve speed and accuracy, those measurements would ideally be automated.

In this study, we hypothesize that it is possible to take full advantage of electronic images availability by devising computer‐aided tools to automate the checks currently included in the QA procedure. In our approach, selected filters specific to the image processing field, such as features detection and geometrical modeling algorithms, are adapted to detect phantom characteristics in the OBI images. The result is a fully automated QA procedure that does not require any user intervention. While retaining the tests, philosophy, and tolerance level established in manual QA protocols, the automated procedure significantly simplifies the QA procedure. The computer‐aided method improves accuracy by eliminating the subjectivity associated with manual interpretation of medical images and provides an “all‐in‐one” approach ideally suited for routine clinical use.

## II. MATERIALS AND METHODS

### 2.1 Phantoms

Most IGRT QA procedures use a phantom that contains a small, well‐defined, central radio‐opaque structure to verify the mechanical accuracy of the OBI system. Different vendors have proposed phantoms that add additional testable features to this basic requirement. Algorithm design and performance on some of the most commonly used phantoms is presented in this section.

**Figure 2:**
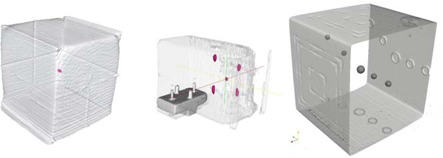
Example of our automated feature extraction tool applied on four different phantoms. (a) The Varian cube phantom consists of a central 2 mm marker embedded in a cube. (b) The Exact Track phantom consist of five radioopaque markers arranged in a star pattern. (c) The Modus phantom consists of five spheres of different sizes embedded in a acrylic cube.

One test phantom is the Varian cube (Varian Medical Systems, Palo Alto, CA) that consists of one central radio‐opaque marker (2 mm diameter) embedded in tissue‐equivalent material. The phantom allows measurement of isocenter deviation by assessing central marker location in the OBI images and checks of image scaling by comparing cube size in the acquired images with their real size. A representation of the phantom's surface and central marker as detected in the CBCT image by our automated procedure is shown in Fig. 2(a).

The Varian Exact T Look‐Bar (MedTec, Orange City, IA) consists of five markers embedded in a tissue‐equivalent material geometric phantom. This phantom is mounted on the couch with a cam lock locking mechanism incorporating a micrometer positioning tool for precisely moving the phantom in lateral, longitudinal, and vertical directions. The phantom consists of a central radio opaque marker placed at isocenter and four other markers distributed around the central one to test orientations and distances. This phantom as viewed by our system is shown in Fig. 2(b).

The Penta‐Guide phantom (Modus Medical Devices, London, Ontario, Canada) consists of an acrylic cube of 16 cm size, with five internal air pockets oriented in a unique pattern (Fig. 2(c). Similar to the other phantoms, internal markers location and size are used to detect correct OBI calibration.

### 2.2 Automated Phantom Feature Detection

#### 2.2.1 Software Platform

A custom software tool was developed in C/C++ to analyze the input images for a complete IGRT QA process using Windows PC platform. The software is based on using “The Visualization Toolkit (VTK)” public domain imaging filters library,^(6)^ which is a collection of hundreds of filters for image processing, mesh generation and manipulation. In this section, the selection of image filters to reproduce the QA procedure is described for the phantoms tested. Although this work is based on the imaging library, the filters used can be found in most image processing software. Our implementation runs automatically all the tests included in the standard QA procedure in less than 1 minute, saves the results in a database, and produces printer‐ready reports of current test results.

Features relevant to the QA procedure are the phantom markers and embedded objects. These features are detected with a succession of segmentation filters to identify phantom objects in the input images. A labeling filter then manipulates objects detected by the segmentation filter.

#### 2.2.2 Segmentation

**Figure 3:**
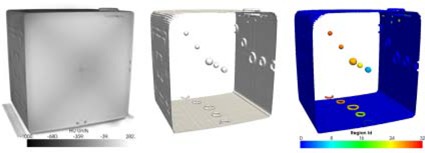
Effect of different image analysis filters used to automate the QA process. The first picture (a) shows a volumetric representation of the CT scan of a Modus phantom. Phantom features (b) are detected using a contouring algorithm with a HU threshold of –500. To select individual features, a labeling algorithm is used. The output of the algorithm is color‐coded in (c), where each individual phantom feature is represented using a different color.

The goal of segmentation is to identify objects in an image by generating isosurfaces of voxels in the input images having intensities above a user‐specified value. Our approach uses the segmentation to identify the phantom markers in the acquired image set which can be kV, MV or CBCT images. To perform the segmentation, we selected the implementation based on the marching cube algorithm because of its speed and accuracy.^(7)^ The only parameter of the contouring filter is the voxel intensity of the markers to be detected, which is phantom‐specific in our implementation. Output of a contouring algorithm applied on a Modus phantom is presented in Fig. 3(b).

#### 2.2.3 Labeling

To further discern between individual objects, a connected components labeling filter is used. Given a segmented image containing objects and the connectivity information between them, the connected component labeling seeks to assign a unique label to each subset of objects that are connected. The output of a labeling algorithm is an image with the same size and spacing as the input image, with each voxel assigned a label according to its associated object. The labeling filter has different modes of operation; the output can be either the labeling of all objects in the input image or the object closest to a given point. Example of a labeling filter applied to distinguish regions within an automatically extracted contour is presented in Fig. 3(c).

### 2.3 Implementation of Specific QA Procedures

Using the image filters described above, we have developed combinations of filters that automate the tasks described in the standard QA protocol by mimicking user interaction and judgment.

#### 2.3.1 Isocenter Accuracy

This QA test evaluates whether the imaging isocenter coincides with the machines dosimetric isocenter. In the typical procedure, CBCT, kV and MV images of the phantom aligned at isocenter are acquired and correct positioning of either kV, MV or CBCT images is checked by calculating the central marker position displacement from the isocenter as observed in the OBI‐acquired images. Ideally, the central marker in all images should be located at isocenter.

To reproduce this test, the segmentation procedure is applied on the input images to detect the phantom markers. If the phantom contains more than one marker, the labeling filter is applied to identify the central marker. Once the central marker is detected, its center is compared with the known isocenter position.

#### 2.3.2 Magnification Accuracy

This test checks image magnification by measuring correct scaling of objects in OBI images. This is done by calculating distances between pairs of markers extracted from the CBCT dataset. The detected values are then compared against phantom specifications.

To implement this test, the segmentation algorithm is used to detect all markers in the input images. The next step executes the labeling algorithm that identifies individual markers near their expected locations. Once all markers' positions are identified, distances between them are calculated to measure distances as reported in the input images. These distances are then compared with the phantom specifications.

#### 2.3.3 OBI Isocenter Accuracy With Gantry Rotation

This test verifies stability of the kV isocenter accuracy as the function of gantry rotation. In the standard QA approach, the phantom is placed on the treatment couch and kV images were acquired at gantry angles 0°, 90°, 180°, and 270° to detect the displacement of the central marker from the digital graticule.

In our implementation, we take advantage of the availability of 600kV‐700kV projections saved during a normal CBCT acquisition procedure which gives a resolution of approximately 0.6° for this procedure as observed in the kV projections acquired for CBCT reconstruction. The location of the central marker in each projected kV, corresponding to a different gantry angle, is obtained and its displacement from the isocenter is calculated.

To implement this test, a region of interest filter is applied on each of the projected CBCT to isolate the central marker, followed by a segmentation procedure to detect marker borders and detection of its center from the borders segmentation. The central marker displacement on each axis is automatically recorded and plotted against gantry angle for comparison.

#### 2.3.4 CBCT Image Quality

The aim of the image quality checks is to verify the HU, contrast, spatial resolution, and HU uniformity of the CBCT images are within acceptable values. This test is specific to the Catphan Phantom (The Phantom Laboratory, Salem, NY) as it uses features available only in this phantom type.

In the manual QA approach, the HU linearity is checked by verifying the HU values of several phantom materials of different densities in the acquired images versus their real values specified in the phantom's documentation. For this purpose, we use the CTP 486 module that is a uniform disk of 20 cm diameter containing different circular regions of uniform HU values. The HU uniformity for each circular insert is measured manually using the area profile tool available in the vendor's software package.

In our implementation, HU linearity is measured by detecting all inserts of the phantom's CTP 486 module. A specific insert is identified using the labeling filter. Statistics of the HU uniformity within one particular insert is obtained using a region of interest to select the insert's core, followed by a histogram filter to display results.

The spatial resolution (high‐contrast resolution) is measured on a special insert that contains bar patterns between 1 to 21 line pairs/cm. The least discernable bar pattern in the CBCT images is identified with the constraint that one should be able to see all five bars in the pattern to count that pattern as discernable.

To implement the spatial resolution test, regions of interest are used to select each pattern, followed by a segmentation algorithm that detects visible bars within each pattern. The next step executes the labeling algorithm that returns the number of detected bars in each pattern. The test on a particular pattern fails if the number of detected bars is less than the real bar count in CTP 528 module. This occurs when neighboring bars became indiscernible due to image degradation.

## III. RESULTS

An example case was created for these geometric QA tests with a plan containing a few anterior‐posterior and lateral kV and MV setup fields, as well as a CBCT field. Images for each of the phantoms described in the previous section were acquired and analyzed using our custom software. Results are reported in this section.

### 3.1 Isocenter Accuracy

**Figure 4:**
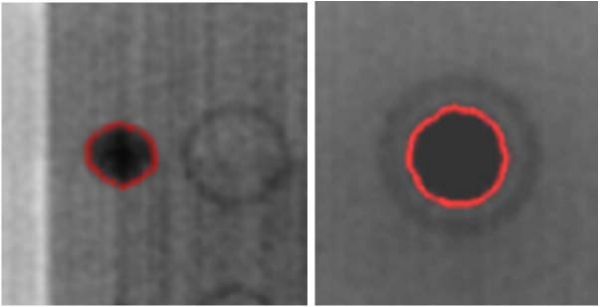
Central marker extraction for the Modus phantom in the kV(left) and MV(right) OBI images. The auto‐extracted contour, represented in red, closely matches marker borders.

**Figure 5:**
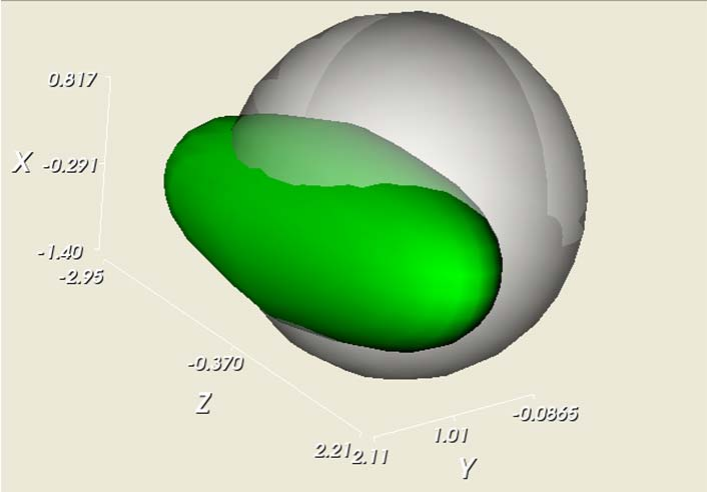
Comparison of the expected (white) and actual (green) marker location for the Varian cube (left) and Modus (right) phantoms. The axes and the corresponding labels are used to measure deviation of the actual marker position from the expected location.

Contour extraction for the Modus phantom from the kV and MV OBI‐acquired images is presented in Fig. 4. For both types of images, a good correspondence is observed between the extracted contour and the background image. Similar results have been obtained for all phantoms. The large HU difference between the marker's voxel intensities and the background values permitted reliable usage of simple, voxel‐based contouring algorithms. The values for the segmentation algorithms are 70% of the maximum intensity for the Varian and Exact T Look‐Bar phantoms, and –500 HU for the Penta‐Guide phantom. The center of the extracted contour is used to measure the OBI's shift. Similarly, a typical isocenter shift assessment using the central marker in the CBCT images is presented in Fig. 5. For comparison purposes, the white wireframe represents the expected marker location and size as specified in the phantom documentation, while the green translucent surface represents the central marker extracted from the CBCT images using the segmentation algorithm. The white scale is used to visualize shift from isocenter, but the software itself automates this measurement by computing the discrepancies between the centers of the white and green contours. A 3D display is implemented such that it can be rotated and zoomed as an alternative tool to inspect results produced by the QA procedure. Fig. 3 provides a representation for each phantom's features extracted from the CBCT datasets.

### 3.2 Magnification Accuracy

Phantom‐specific distances in the OBI and CBCT are detected and computed using the contouring and labeling algorithm. For the Varian phantom, the size of the central marker is used. For the Varian Exact T Look‐Bar and Modus phantoms, the distances between paired phantom markers are used.

### 3.3 OBI Isocenter Accuracy With Gantry Rotation

**Figure 6:**
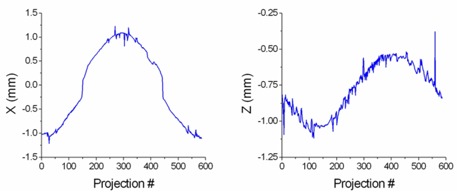
Winston‐Lutz tests are performed directly on the OBI‐acquired projections of a CBCT scan. The gantry sag with rotation on the X and Y directions is plotted in these graphs.

Isocenter localization with gantry rotation of the Varian phantom (using the 625 projections spanning 360° acquired during CBCT acquisition) is presented in Fig. 6. A region of interest (1 cm square side) centered on the central marker was used to improve the speed and stability of the algorithm. Since the intensity of the central marker varies as the gantry rotates, a value of 60% of the maximum intensity in the ROI region was used by the segmentation algorithm. To eliminate noise, the labeling algorithm was then configured to select the largest object returned by the segmentation algorithm. Results shown in Fig. 6 demonstrate that, as the gantry rotated, the isocenter was stable within 2 mm for the X direction and 1.5 mm for the Z direction. The noise‐like signal at different gantry angles was attributed to the contouring algorithm's pickling noise in the kV images. It is interesting to note that in all 625 projections, the error introduced in the contouring by the inherent noise did not affect the contouring algorithm by more than 1 mm.

## IV. DISCUSSION

The focus of the proposed QA procedure is to automate a medical physicist's work verifying if current machine parameters are within the tolerances specified in the protocol. For this purpose, an automated image analysis framework was devised and tested. Phantom features are of sufficient contrast to permit reliable usage of simple, threshold‐based contouring filters, without any user interaction. A labeling algorithm was used to classify extracted phantom features. Based on the features present in the phantom used, different geometrical or voxel‐based checks can be devised to verify the OBI accuracy. The segmentation, labeling and measurement filters did work reliably on all tested phantoms.

Previous guidelines for CBCT QA have suggested measurement of the machine's mechanical parameters such as detector‐to‐isocenter and source‐to‐isocenter distances. These are difficult and time‐consuming as the detector and source covers have to be removed. Our view is that these are unnecessary measurements since it is unclear what error magnitude would produce a significant deviation in image quality. Additionally, it is not known which mechanical parameters significantly affect the machine's geometrical stability. The proposed QA procedure does not assume a priori information on the mechanical parameters that will influence the accuracy of the OBI system. As compared to standard protocols, the proposed QA procedure takes direct advantage of the available OBI capabilities to replace measurements of some mechanical parameters affecting image quality (such as source‐to‐isocenter and source‐to‐detector) with direct interpretation of phantom features observed in the onboard images. Any mechanical calibration error that will influence image parameters will be detected by the location and size checks, and reported by our revised procedure.

On the four linear accelerator machines tested in our institution over a one‐year period, the coincidence between the CBCT radiation and imaging isocenter was found to be within 2 mm for all four accelerators. Both the kV and MV imaging was in within 1.5 mm accuracy. Isocenter stability with gantry angle was within 1 mm. Results obtained with the automated protocol were consistently stable and similar to the results reported by other groups.^(4)^

The automated protocol implementation retains the phantoms and methods in use today as well as new tests. The automated image interpretation test allows verification of isocenter displacement for every half degree. Additionally, the availability of electronic results permitted development of convenience tools in our software such as automated reporting, saving to database, and trends analysis. Because it relies directly on the acquired images and it does not use any OBI‐related software, our tool constitutes an independent check of the vendor software.

Parameters are measured directly on the acquired images, providing a faster and simpler protocol that is free of human error as compared with the standard procedures. With this approach phantom setup on the couch is the only procedure subject to human errors, and there are no multiple human‐defined variables in the protocol that may cancel out to give an acceptable result when an error exists. All imaging modalities available on the OBI system are analyzed. These include: (a) a volumetric CBCT image of the phantom situated at isocenter, (b) the projected kV images used for reconstructing the CBCT volume, (c) a set of MV images acquired at different gantry angles, and (d) a set of kV images acquired at different gantry angles.

## V. CONCLUSIONS

In this paper, we have presented a computer‐aided automated quality assurance method for the OBI system. The central idea is that the features necessary for the QA procedure can be automatically detected in acquired images using a combination of image filtering algorithms to establish a reliable comparison between the measured and expected machine parameters. Different from other type of QA protocols that use DRRs and image registration, our method directly measures features in the acquired images and thus is immune to human or registration errors. Compared to the conventional QA approach, the automated procedure is significantly faster and more robust as it uses fully objective and quantitative measurements. Our study demonstrated that the information contained in the kV, MV and CBCT images is sufficient to guide the contouring filters reliably without any user interaction. The proposed protocol decreases the workload involved in OBI QA and provides a valuable tool for the efficient use of available onboard information for adaptive treatment planning.
